# Anatomic Variants of the Anterior Cerebral Artery and Anterior Communicating Artery: A Digital Subtraction Angiographic Study

**DOI:** 10.7759/cureus.104548

**Published:** 2026-03-02

**Authors:** Anjali K Mathew, Rathi Sudhakaran, Srikanth Moorthy

**Affiliations:** 1 Anatomy, Government Medical College, Ernakulam, Kochi, IND; 2 Anatomy, Amrita School of Medicine, Amrita Institute of Medical Sciences, Kochi, IND; 3 Radiology, Amrita School of Medicine, Amrita Institute of Medical Sciences, Kochi, IND

**Keywords:** anatomic variants, aneurysm, anterior cerebral artery, anterior communicating artery, digital subtraction angiography

## Abstract

Background

Several anatomic variants can result from developmental anomalies of the cerebral arteries during the embryonic period. Some of them predispose to the development of aneurysms or cerebrovascular ischemic disease. Failure to identify them may lead to misdiagnosis. This study aimed to document the frequency and type of anatomic variants of the anterior cerebral artery (ACA) and the anterior communicating artery (ACoA).

Methodology

A retrospective, record-based, cross-sectional study was conducted among 500 subjects (188 females, 312 males) whose four-vessel digital subtraction angiographic images of the ACA and ACoA were reviewed for the presence of anatomic variants such as aplasia, hypoplasia, azygous ACA, fenestrations, duplications, and associated aneurysms.

Results

Among the total 500 images evaluated, anatomic variants of the ACA were present in 3.6%. The most common variant was aplasia of the A1 segment of the ACA (2%), followed by hypoplasia of the A1 segment of the ACA (1.4%) and azygous ACA (0.2%). No anatomic variants were seen in the ACoA. Associated aneurysms were noted in 4.2% of subjects, among which 3.2% were seen in the ACoA and 1% in the distal ACA. Among the aneurysm cases associated with anatomic variants, six were associated with aplasia of the A1 segment of the ACA, one with hypoplasia of the A1 segment of the ACA, and one with azygous ACA.

Conclusions

Anatomical variations of the ACA were seen in less than 5% of the study population. The most common anatomic variants were aplasia and hypoplasia of the A1 segment of the ACA, followed by azygous ACA. Few aneurysms were seen, the majority of which were in the ACoA. Some of the aneurysms noted were associated with anatomic variants. Presence of vascular variations of the ACA and ACoA can be asymptomatic and uncomplicated, but knowledge and identification of them may help neurosurgical planning and prevent medical errors.

## Introduction

The brain is a vital organ that depends on a continuous and well-regulated blood supply to maintain normal function [1]. Cerebral circulation is broadly divided into the anterior circulation, supplied by the internal carotid arteries, and the posterior circulation, supplied by the vertebrobasilar system [2-4]. The anterior circulation primarily supplies the forebrain, while the posterior circulation supplies the occipital lobes, cerebellum, and brainstem [3,4].

The anterior cerebral artery (ACA), a terminal branch of the internal carotid artery, forms an essential component of the anterior circulation. The paired ACAs are connected by the anterior communicating artery (ACoA), constituting the anterior portion of the circle of Willis and contributing to collateral cerebral perfusion [2,4].

Anatomical variations in the ACA-ACoA complex are frequently encountered in a substantial proportion of both cadaveric and angiographic studies [5]. These variations, termed anatomic variants, are primarily attributed to developmental anomalies of cerebral arteries occurring during the embryonic period. They include aplasia, hypoplasia, azygous ACA, duplication, fenestrations, and persistent fetal arteries [4]. These variants of the cerebral arteries are often clinically silent but may acquire greater importance in the presence of cerebrovascular pathology [6].

The anterior circulation is recognized as the most common site of intracranial aneurysms, with a significant proportion of these lesions arising from the ACA and ACoA [6,7]. Previous studies have suggested a possible association between anatomical variants of the ACA-ACoA complex and aneurysm formation. This anatomical information is crucial for the accurate interpretation of angiographic studies and for safe planning and management of neurosurgical and endovascular interventions, particularly in patients presenting with ischemic stroke or subarachnoid hemorrhage [6,7].

Available Indian literature on angiographic variations of the ACA and ACoA is limited. Hence, this retrospective observational digital subtraction angiographic (DSA) study aimed to identify and document anatomical variants of the ACA and ACoA in an Indian population based on angiographic analysis.

## Materials and methods

Study design and subjects

This retrospective, record-based, cross-sectional study was performed at the departments of Anatomy and Radiology of Amrita Institute of Medical Sciences, Kochi, India. Ethical clearance was obtained from the Institutional Ethical Committee of Amrita Institute of Medical Sciences and Research Centre (approval number: dissertation review/MD/MS/2011, approval date: 4/10/2011). Four-vessel DSA images of 500 patients who had attended the radiology department from 2007 to 2013 were collected using the institution’s Amrita MedVision software and evaluated retrospectively. Angiograms demonstrating a normal course and morphology of the ACA and ACoA were included in the study. Images with pathology that significantly altered or obscured the normal anatomical course of the ACA-ACoA complex, such as occlusion, infarction-related distortion, ruptured aneurysms, or prior surgical or endovascular interventions, were excluded. Unruptured aneurysms were included, provided that the ACA and ACoA anatomy was preserved and could be reliably evaluated. Images were analyzed irrespective of age, sex, and the presence of vascular pathology affecting other intracranial arteries.

Digital subtraction angiography

DSA is a fluoroscopic imaging technique that enables detailed visualization of intracranial vasculature by digitally subtracting pre-contrast images from post-contrast images. It is considered the reference standard for evaluating cerebral arterial anatomy. Images are acquired at a rate of 1-6 frames per second following intra-arterial contrast administration, allowing high-resolution assessment of the ACA and ACoA [8].

Imaging evaluation and anterior cerebral artery segmentation

All DSA images were acquired according to the institution’s routine clinical protocol, including standard femoral artery access, routine projection views, and standard contrast injection parameters. Specific procedural details were not assessed, as this was a retrospective anatomical study. The anteroposterior, lateral, and oblique views of DSA images of the ACA and ACoA were reviewed. The images were interpreted with the help of an experienced radiologist specialized in neurovascular imaging.

ACA segmentation was performed according to standard neuroanatomical and angiographic descriptions, dividing the ACA into five segments (A1-A5). The A1 segment was defined as the pre-communicating segment extending from the internal carotid artery bifurcation to the ACoA; the A2 segment as the post-communicating segment distal to the ACoA; and the A3 segment as the precallosal segment curving around the genu of the corpus callosum. The A4 (supracallosal) and A5 (postcallosal) segments represent the distal pericallosal course. The A1 and A2 segments represent the proximal ACA, whereas A3-A5 segments represent the distal ACA [4,5].

Predefined operational criteria were used to classify anatomical variants of the ACA-ACoA complex based on DSA. A1 segment hypoplasia was defined as a reduction in vessel caliber greater than 50% compared with the contralateral A1 segment. A1 segment aplasia was defined as complete non-visualization of the A1 segment. An azygous ACA was defined as a single dominant A2 trunk supplying both cerebral hemispheres [5]. Variants were recorded only when clearly visualized in more than one angiographic projection.

Statistical analysis

Data were analyzed using descriptive statistical methods. The frequency and percentage of each anatomical variant of the ACA-ACoA complex were calculated. To estimate the precision of the observed proportions, 95% confidence intervals (CIs) were computed for each variant using standard formulas for proportions, with the total sample size fixed at 500. All statistical analyses were performed using Microsoft Excel (Microsoft Corp., Redmond, WA, USA). Given the retrospective and descriptive nature of the study, no inferential statistical tests or analyses of association were undertaken.

## Results

A total of 500 DSA images of the ACA and ACoA were evaluated in this study. The participants ranged in age from 4 to 75 years (mean age = 48.9 ± 16.5 years), representing both pediatric and adult populations. Of the total images analyzed, 62.4% were obtained from male participants and 37.6% from female participants.

Observed anatomic variants

**Figure 1:**
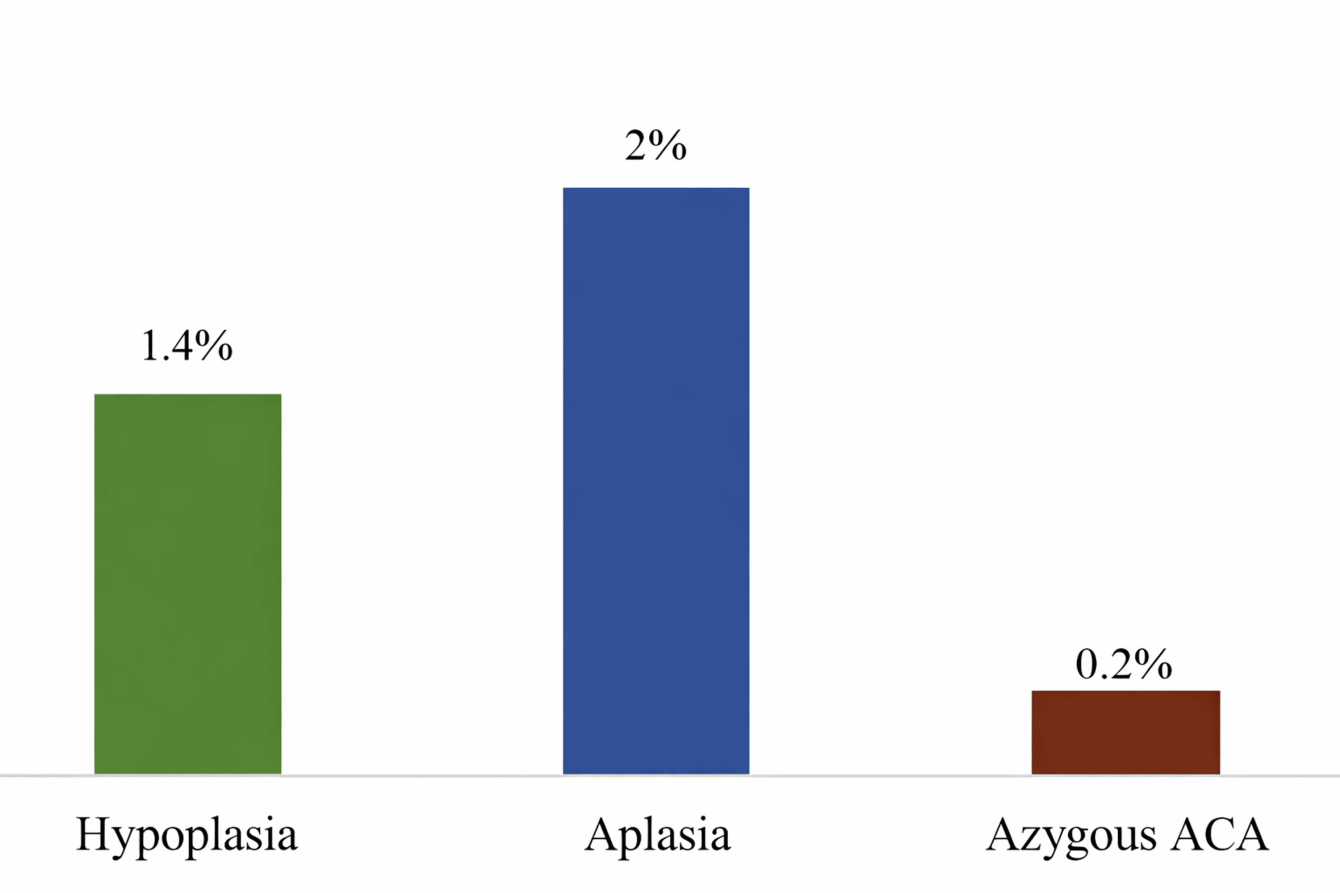
Distribution of anatomic variants. ACA = anterior cerebral artery

Anatomical variations of the ACA were noted in 3.6% of the study population. The most prevalent anatomic variants found were aplasia of the A1 segment of the ACA (2%), followed by hypoplasia of the A1 segment of the ACA (1.4%) and azygous ACA A2 segment (0.2%). No anatomical variations of the ACoA were seen. No duplicated or fenestrated ACA or ACoA were seen. Table 1 and Figure 1 show the distribution of anatomic variants noted in the study.

**Table 1:** Distribution of anatomic variants. ACA = anterior cerebral artery; CI = confidence interval

ACA	Cases with variation	Percentage	95% CI
Hypoplasia	7	1.4	0.6–2.9
Aplasia	10	2	1.0–3.6
Azygous ACA	1	0.2	0.1–1.1
Duplications	0	0	0–0.7
Fenestration	0	0	0–0.7

**Figure 2:**
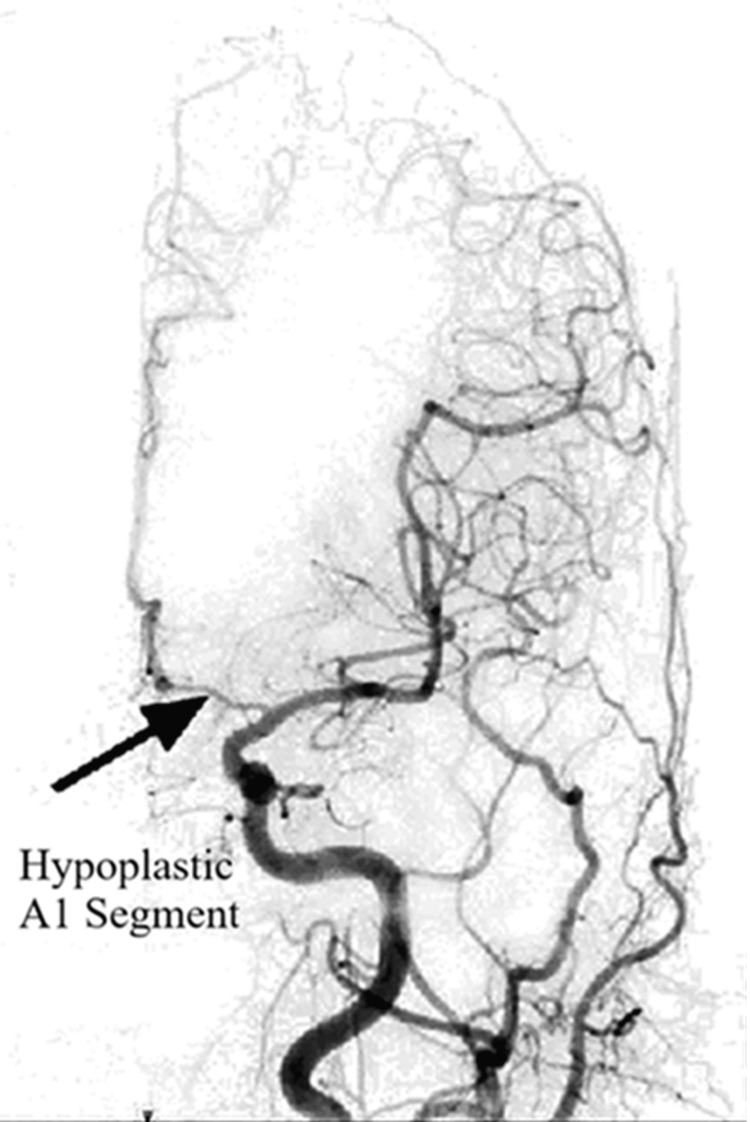
Anteroposterior view showing the hypoplastic A1 segment on the left side. The figure is derived from digital subtraction angiographic images of the patients included in the current study.

Of the 500 cases examined, seven (1.4%) had hypoplasia of the A1 segment of the ACA, of which five were in males and two were in females. Three cases had hypoplasia on the right side, while four cases had hypoplasia on the left side (Figure 2). No cases of hypoplasia were noted in the A2 segment of the ACA or ACoA. Table 2 presents the distribution of hypoplasia.

**Table 2:** Distribution of hypoplasia. ACA = anterior cerebral artery; ACoA = anterior communicating artery

Hypoplasia	ACA		ACoA, n (%)
	A1, n (%)	A2, n (%)	
Present	7 (1.4)	0 (0)	0 (0)
Absent	493 (98.6)	500 (100)	500 (100)
Total	500	500	500

**Figure 3:**
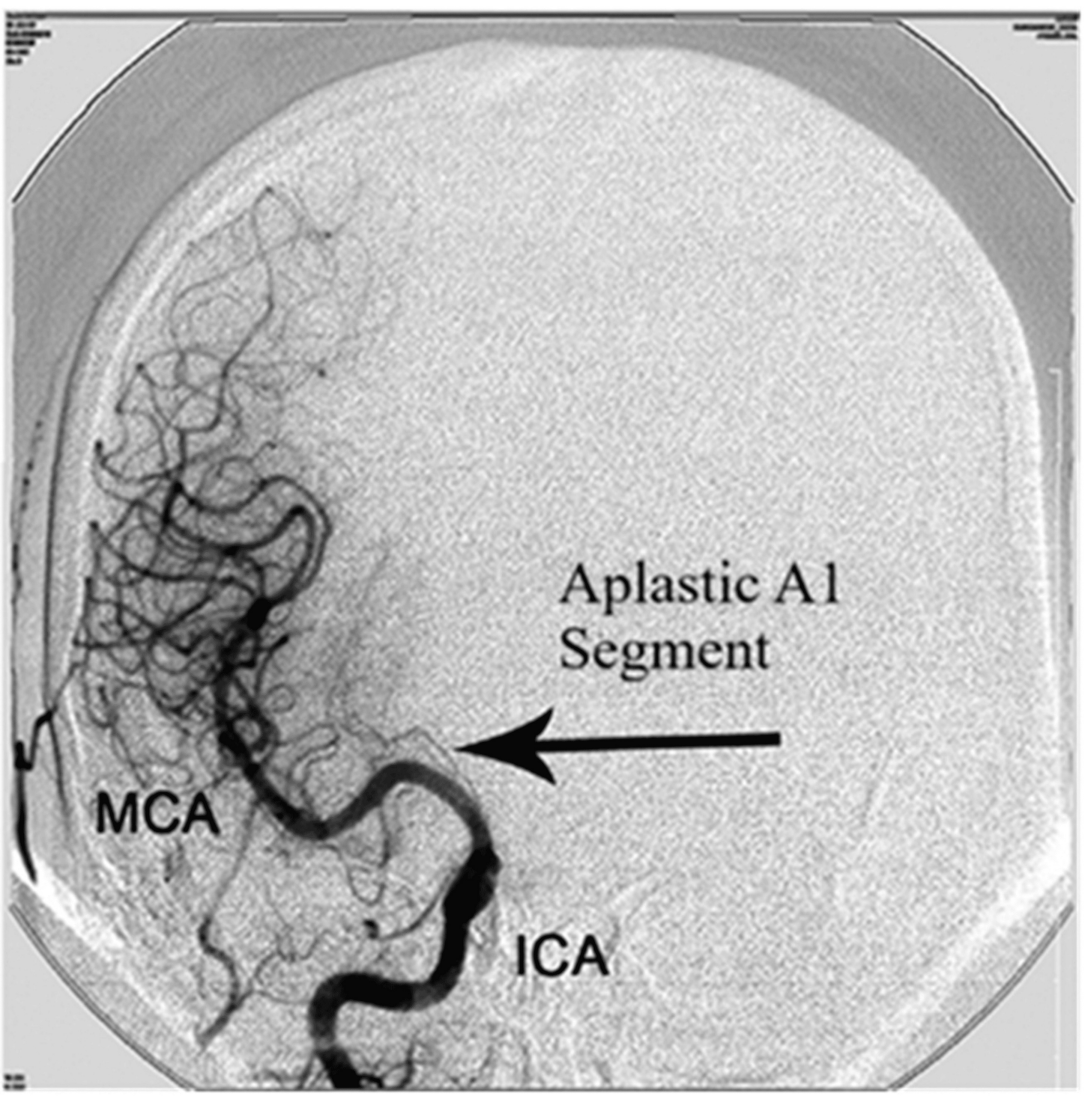
Anteroposterior view showing the aplastic A1 segment on the right side. The figure is obtained from digital subtraction angiographic images of the patients included in the current study. MCA = middle cerebral artery; ICA = internal carotid artery

Among the total 500 cases, 10 (2%) had aplasia of the A1 segment of the ACA, of which seven were noted in males and three in females. All cases were on the right side (Figure 3). No aplasia was detected in the A2 segment of the ACA or ACoA. Table 3 shows the distribution of aplasia.

**Table 3:** Distribution of aplasia. ACA = anterior cerebral artery; ACoA = anterior communicating artery

Aplasia	ACA		ACoA, n (%)
	A1, n (%)	A2, n (%)	
Present	10 (2)	0 (0)	0 (0)
Absent	490 (98)	500 (100)	500 (100)
Total	500	500	500

**Figure 4:**
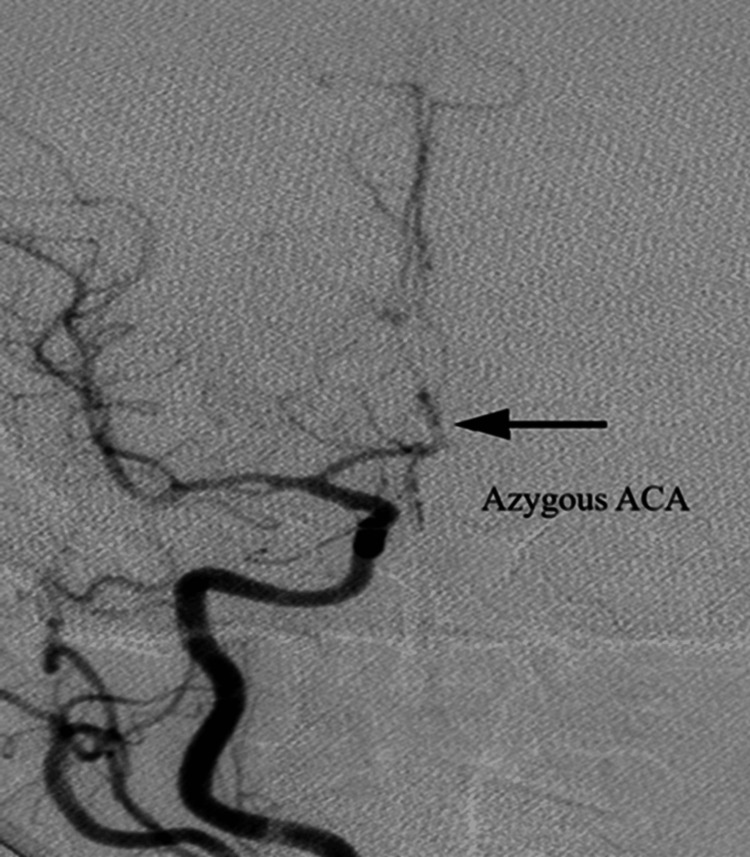
Anteroposterior view showing an azygous ACA. The figure is obtained from digital subtraction angiographic images of the patients included in the current study. ACA = anterior cerebral artery

Of the total 500 cases, an azygous A2 segment of the ACA was noted only in one female patient (Figure 4).

Anterior cerebral artery

**Figure 5:**
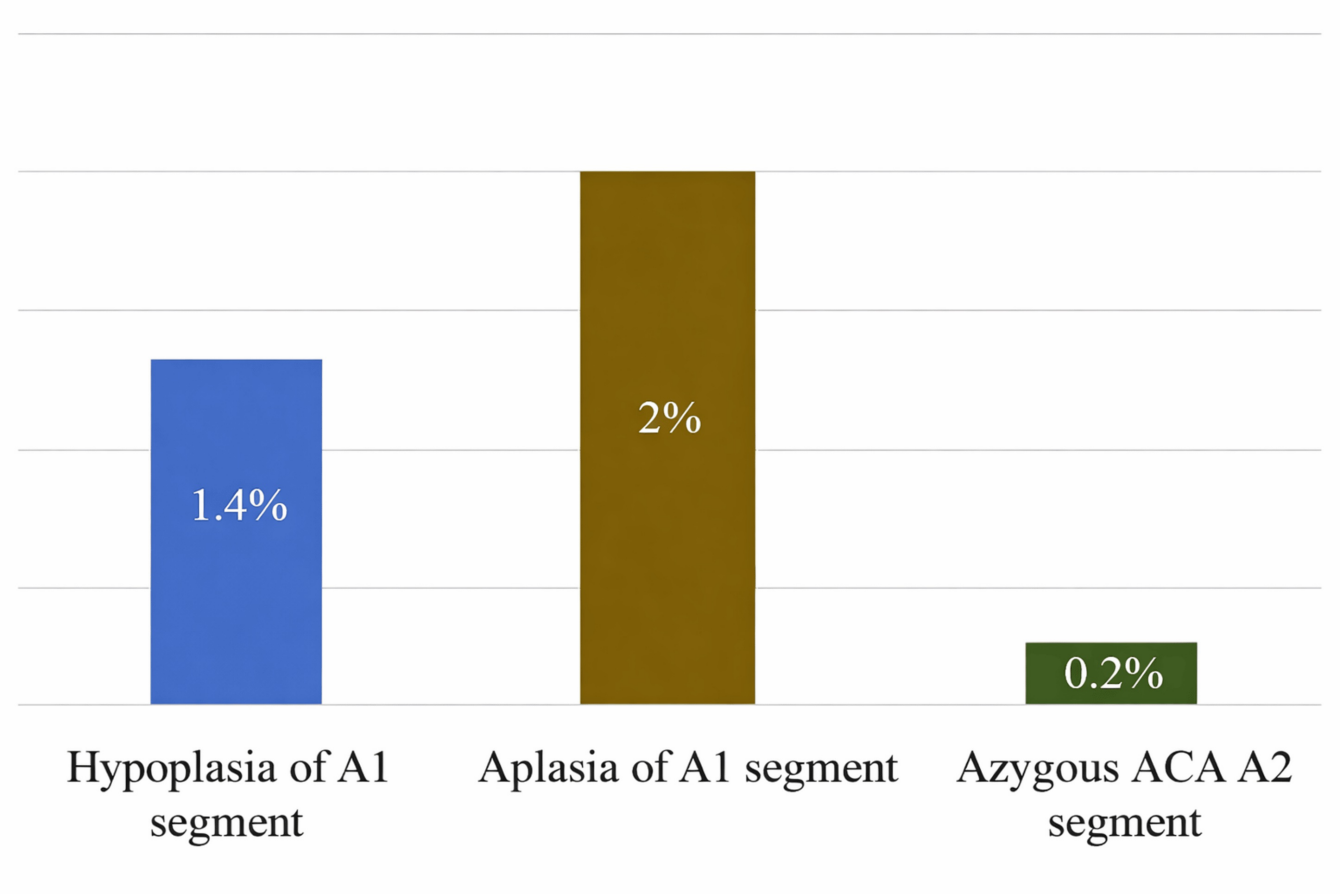
Anatomic variants in the ACA. ACA = anterior cerebral artery

The typical form of this vessel was found in 96.4% of the study subjects. Among the anatomic variants in the ACA, the most common in this study was aplasia in the A1 segment (2%), followed by hypoplasia in the A1 segment (1.4%), and azygous ACA in the A2 segment (0.2%) (Figure 5). No duplication or fenestration was seen in the ACA.

**Figure 6:**
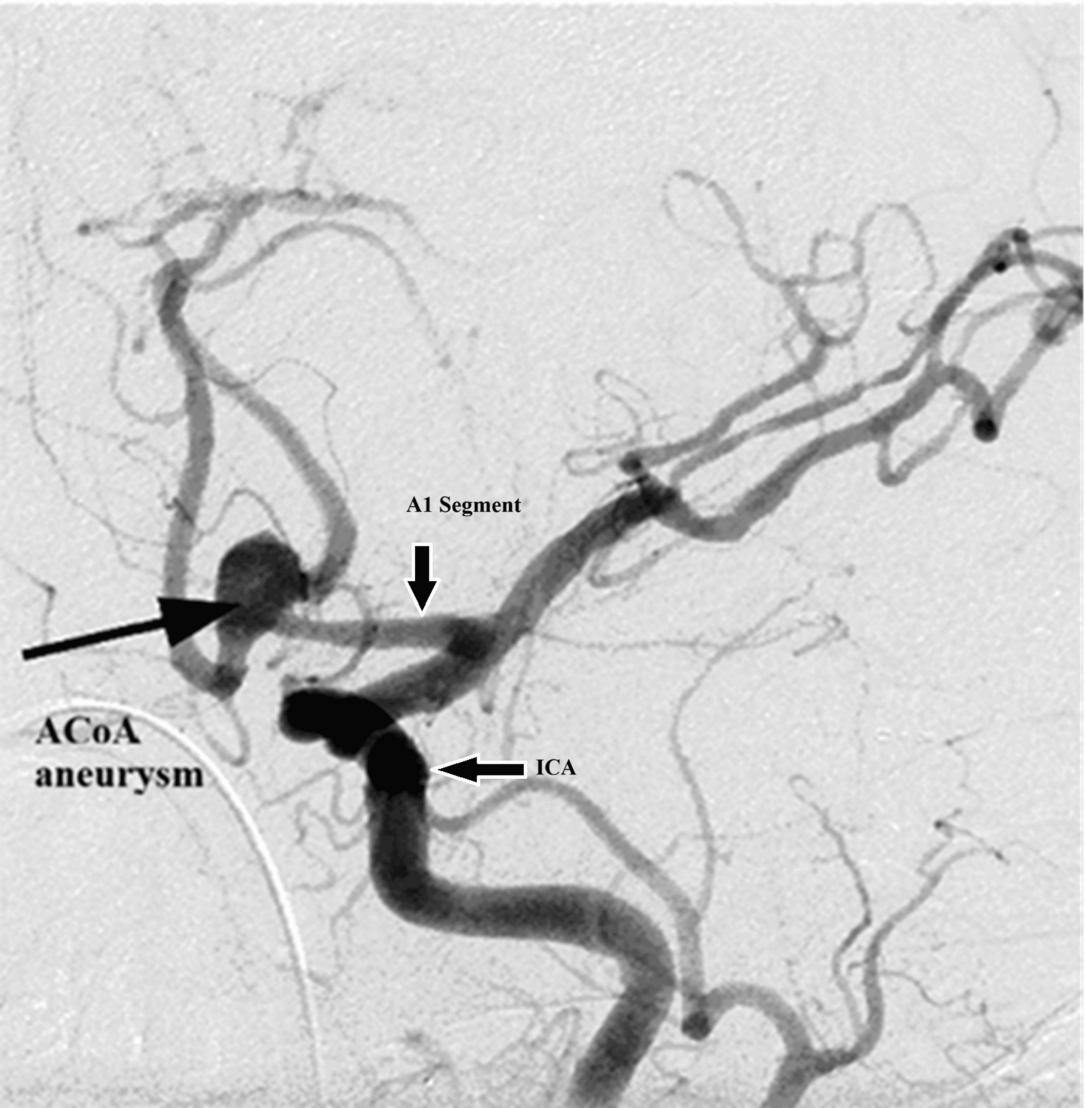
Oblique view of an aneurysm on the ACoA. The figure is obtained from digital subtraction angiographic images of the patients included in the current study. ACoA = anterior communicating artery; ICA = internal carotid artery

**Figure 7:**
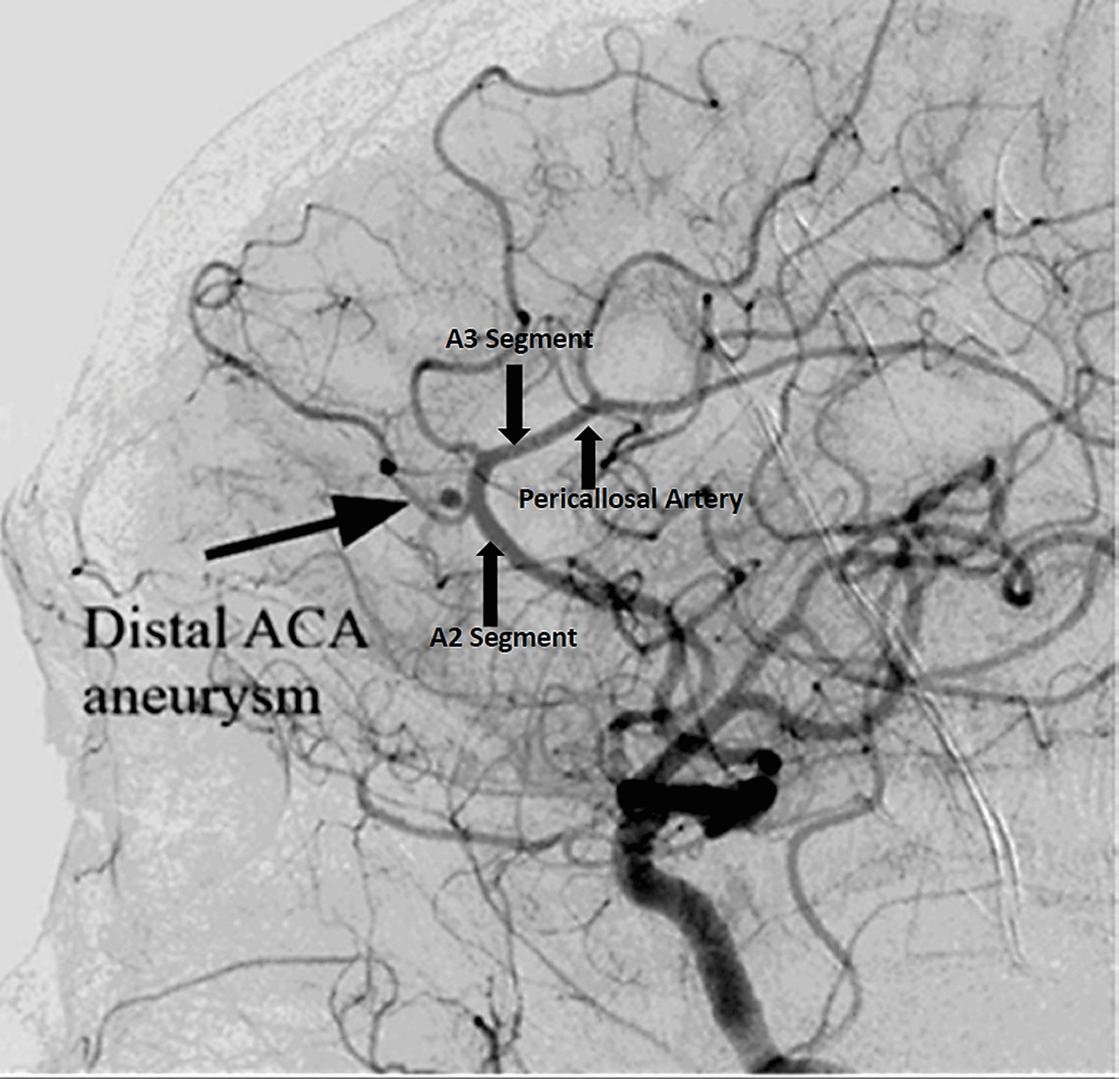
Lateral view of a distal ACA aneurysm (junction of the A2 and A3 segments). The figure is obtained from digital subtraction angiographic images of the patients included in the current study. ACA = anterior cerebral artery

The typical anatomy of the ACoA was observed in all cases, and no anatomic variants were identified. Associated aneurysms were noted in 4.2% of the study subjects (21 cases). Among these, 3.2% of aneurysms were located at the ACoA (16 cases) (Figure 6), while 1% (5 cases) were identified in the distal ACA (junction of A2 and A3 segments) (Figure 7) or the A3 segment.

Association of aneurysms with anatomic variants

Of the 16 cases of aneurysm in ACoA, six were associated with aplasia in the A1 segment of the ACA. Of the five cases of aneurysm in the ACA, one was associated with hypoplasia of the A1 segment of the ACA, and one was associated with azygous ACA. Thus, a total of eight cases of aneurysms were seen along with variants, whereas in the rest of the 13 cases, no variants were detected (Table 4).

**Table 4:** Distribution of cases of aneurysms in association with the anatomic variants. ACA = anterior cerebral artery; ACoA = anterior communicating artery

Arteries	Cases of aneurysm	Number of cases with anatomic variants			Number of cases without variants
		Hypoplasia	Aplasia	Azygous	
ACoA	16	-	6	-	10
ACA	5	1	-	1	3

## Discussion

Several researchers have performed studies on the ACA and ACoA worldwide by gross dissection and angiographic techniques. The anatomic variants of the ACA and ACoA include aplasia, hypoplasia, duplication, fenestration, and azygous ACA. The present study aimed to document the incidence of these anatomic variants by analyzing DSA images of the ACA and ACoA in 500 subjects who had attended the Department of Radiology of Amrita Institute of Medical Sciences, Kochi.

Anatomical variations were present in 3.6% of the study population. The predominant anatomic variant noted was aplasia of the A1 segment of the ACA (2%), followed by hypoplasia of the A1 segment of the ACA (1.4%) and the azygous ACA A2 segment (0.2%). No anatomic variants of the ACoA were seen. Medical literature has identified agenesis and hypoplasia as anatomical factors associated with defective circulation. Unilateral A1 agenesis or aplasia has been reported in different populations by different researchers. Studies from cadaveric dissection by Puchades-Orts et al. [9] reported 0.8% cases of aplasia of the A1 segment of the ACA in the Spanish population, Saikia et al. [10] from India reported 2% cases of aplastic A1 segment, and Curry and Culberth [11] from the United States noted one case of aplastic ACA. Uchino et al. [12] reported 5.6% cases of unilateral A1 segment aplasia from MR angiography in the Japanese population. In the present study, aplasia was noted in 2% of cases. All cases were noted on the right side, and of the 10 cases, seven were in males. In the study by Uchino et al. [12], aplasia was more common in males with a higher incidence on the right side of the ACA. Another MR angiographic study by Ješić et al. [13] also reported 2.9% unilateral aplasia, more in males. In this regard, our study findings were similar to the reports of Uchino et al. and Ješić et al. In a study of 1,000 medicolegal autopsy specimens of the brain, Kapoor et al. [14] reported 0.4% aplastic A1 segments, which is much lower than the present study.

Many studies on the occurrence and distribution of A1 segment hypoplasia have been performed in different populations. Puchades-Orts et al. [9] from Spain noted 6.4% hypoplasia of the A1 segment of the ACA. Riggs and Rupp [15] reported 7% cases of hypoplastic A1 segment in Philadelphia. From the Sri Lankan population, De Silva et al. [16] reported 5% cases of A1 segment hypoplasia. Cadaveric studies performed in North India by Saikia et al. [10] reported 7% cases. All these were much higher than the current study prevalence of 1.4%, which was similar to the study by Nordon and Júnior [17] from Brazil, with a prevalence of 2%, Kapoor et al. [14] with 1.7%, and Swetha et al. [18] from South India that reported 1.4% cases of A1 segment hypoplasia. The reported prevalence of azygous ACA in the literature is between 0.2% and 4% [5]. This study reported a prevalence of 0.2%. Baptista [19] reported 1% azygous ACA, Serizawa et al. [20] 3% azygous ACA, whereas Gunnal et al [21] noted 11.6% incidence from Maharashtra.

The present study showed no cases of fenestration or duplication of the ACA. Similar studies have also reported a very low incidence of duplication and fenestration. Uchino et al. [12] noted 1.2% cases of fenestration of the A1 and A2 segments, and Ješić et al. [13] noted 0.5% fenestration of the A1 segment. Swetha et al. [18] from India reported A1 segment fenestration in 1.4%. The normal variants of ACoA have also been reported in the literature. Fenestration of ACoA was reported in 5.8% cases by Siddiqi et al. [22], and duplicated ACoA in 10% of cases by Kapoor et al. [14].

Approximately 90% of all intracranial aneurysms arise on the anterior carotid circulation, of which 30-35% are in the ACoA. In the present study, 21 cases of aneurysm were detected in the ACA and ACoA. Of these, 16 were in the ACoA, and five were in the distal ACA. Of the 16 cases of ACoA aneurysm, six were associated with A1 segment aplasia. Uchino et al. [12] in the Japanese population of 891 reported seven cases of ACoA aneurysms, all with A1 segment aplasia. In a DSA study, Bapuraj et al. [23] from India noted two cases of saccular aneurysm of the azygous ACA. In the present study, only one case of azygous ACA was noted, associated with a distal ACA aneurysm at the ACA bifurcation. Abubakr et al. [24], in a similar study from Sudan, detected two cases of ACoA aneurysms. Agayev et al. [25] from Turkey reported the incidence of A1 segment hypoplasia to be higher in the ACoA aneurysm group. Compared to this, our study showed A1 segment aplasia to be higher in the ACoA aneurysm group.

Limitations

This retrospective, hospital-based study may be subject to selection bias. Imaging was analyzed by a single experienced radiologist without an interobserver reliability assessment, which may limit reproducibility. As the study was descriptive, no inferential statistical analysis was performed, and the findings should be interpreted as descriptive rather than causal or associative.

## Conclusions

In this retrospective DSA-based analysis, the prevalence of anatomic variants of the ACA-ACoA complex was relatively low compared with previously published studies. Variations involving the A1 segment of the ACA, namely, aplasia and hypoplasia, were the most frequently observed, followed by the presence of azygous ACA. No instances of arterial duplication, fenestration, or variations of the ACoA were detected. Aneurysms were most often located at the ACoA, and coexisting anatomic variants were observed in a subset of aneurysm cases. Recognizing the presence and clinical relevance of anatomical variants is important, and the findings of this study may assist neurosurgeons and radiologists in planning and performing neurological and radiological procedures safely. Further prospective studies with larger cohorts are needed to better understand any potential relationship between these variants and aneurysm formation.

## References

[1] Snell RS (2009). Clinical Neuroanatomy.

[2] Molnár Z (2004). Thomas Willis (1621-1675), the founder of clinical neuroscience. Nat Rev Neurosci.

[3] Edvisson L, Krause DN (2002). General & comparative anatomy of the cerebral circulation. Cerebral Blood Flow and Metabolism.

[4] Standring S (2008). Gray’s Anatomy: The Anatomical Basis of Clinical Practice.

[5] Osborn AG (1998). Diagnostic Cerebral Angiography. Philadelphia: Lippintoncott Williams & Wilkins.

[6] JP Mohr, Wolf PA, Grotta JC, Moskowitz MA, Mayberg MR, Von Kummer R (2011). Anterior cerebral artery disease. Stroke: Pathophysiology, Diagnosis & Management.

[7] Shapio M (2025). Neuroangio. Anterior cerebral artery. https://neuroangio.org/anatomy-and-variants/anterior-cerebral-artery/.

[8] Sutton D (2012). Radiology and Imaging for Medical Students.

[9] Puchades-Orts A, Nombela-Gomez M, Ortuño-Pacheco G (1976). Variation in form of circle of Willis: some anatomical and embryological considerations. Anat Rec.

[10] Saikia B, Tuudkar K, Sarma J, Sarma A, Madaan S (2013). Study of variations of the anterior cerebral artery in human brain. Natl J Clin Anat.

[11] Curry RW, Culbreth GG (1951). The normal cerebral angiogram. Am J Roentgenol Radium Ther.

[12] Uchino A, Nomiyama K, Takase Y, Kudo S (2006). Anterior cerebral artery variations detected by MR angiography. Neuroradiology.

[13] Ješić A, Torbica S, Marić S, Popović S, Kozić D (2011). Anatomic variatons of the anterior portion of the circle of Willis: MR angiographic study. Curr Top Neurol Psychiatr Relat Discip.

[14] Kapoor K, Singh B, Dewan LI (2008). Variations in the configuration of the circle of Willis. Anat Sci Int.

[15] Riggs HE, Rupp C (1963). Variation in form of circle of Willis. The relation of the variations to collateral circulation: anatomic analysis. Arch Neurol.

[16] De Silva KR, Silva R, Gunasekera WS, Jayesekera RW (2009). Prevalence of typical circle of Willis and the variation in the anterior communicating artery: a study of a Sri Lankan population. Ann Indian Acad Neurol.

[17] DG Nordon, Júnior R (2012). Variations in the brain circulation: the circle of Willis. J Morphol Sci.

[18] Swetha B, RK Dakshayani (2011). Variable anterior cerebral artery in human cadavers. Anat J Karnataka.

[19] Baptista AG (1963). Studies on the arteries of the brain. II. The anterior cerebral artery: some anatomic features and their clinical implications. Neurology.

[20] Serizawa T, Saeki N, Yamaura A (1997). Microsurgical anatomy and clinical significance of the anterior communicating artery and its perforating branches. Neurosurgery.

[21] Gunnal SA, Wabale RN, Samsamuddin Farooqui MS (2013). Variations of anterior cerebral artery in human cadavers. Neurol Asia.

[22] Siddiqi H, Tahir M, Lone KP (2013). Variations in cerebral arterial circle of Willis in adult Pakistani population. J Coll Phys Surg Pak.

[23] Bapuraj JR, Ojili V, Khandelwal N, Kaza RK, Shanbhogue AK, Chabbra R (2007). Case series: saccular aneurysm of the azygous anterior cerebral artery: report of 2 cases & review of literature. Indian J Radiol Imaging.

[24] Alawad HM, Hussein MA, Hassan MA (2009). Morphology and normal variations of the cerebral arterial circle of Willis in Khartoum Diagnostic Centre. Khartoum Med J.

[25] Agayev K, Onal B, Yavuz K, Ziyal IM (2005). The association of A1 segment hypoplasia/aplasia with anterior communicating artery aneurysms: a radiological study. Turk Neurosurg.

